# Intrinsic brain indices of verbal working memory capacity in children and adolescents

**DOI:** 10.1016/j.dcn.2015.07.007

**Published:** 2015-08-04

**Authors:** Zhen Yang, Devika R. Jutagir, Maki S. Koyama, R. Cameron Craddock, Chao-Gan Yan, Zarrar Shehzad, F. Xavier Castellanos, Adriana Di Martino, Michael P. Milham

**Affiliations:** a Center for the Developing Brain, Child Mind Institute, 445 Park Avenue, New York, NY 10022, USA; b Nathan Kline Institute for Psychiatric Research, Orangeburg, NY 10962, USA; c Department of Psychology, University of Miami, Coral Gables, FL 33146, USA; d The Child Study Center at NYU Langone Medical Center, New York, NY 10016, USA; e Department of Psychology, Yale University, New Haven, CT 06520, USA

**Keywords:** Development, Digit span, Intrinsic brain activity, Resting-state fMRI, Brain–behavior relationships

## Abstract

Working memory (WM) is central to the acquisition of knowledge and skills throughout childhood and adolescence. While numerous behavioral and task-based functional magnetic resonance imaging (fMRI) studies have examined WM development, few have used resting-state fMRI (R-fMRI). Here, we present a systematic R-fMRI examination of age-related differences in the neural indices of verbal WM performance in a cross-sectional pediatric sample (ages: 7–17; *n* = 68), using data-driven approaches. Verbal WM capacity was measured with the digit span task, a commonly used educational and clinical assessment. We found distinct neural indices of digit span forward (DSF) and backward (DSB) performance, reflecting their unique neuropsychological demands. Regardless of age, DSB performance was related to intrinsic properties of brain areas previously implicated in attention and cognitive control, while DSF performance was related to areas less commonly implicated in verbal WM storage (precuneus, lateral visual areas). From a developmental perspective, DSF exhibited more robust age-related differences in brain–behavior relationships than DSB, and implicated a broader range of networks (ventral attention, default, somatomotor, limbic networks) – including a number of regions not commonly associated with verbal WM (angular gyrus, subcallosum). These results highlight the importance of examining the neurodevelopment of verbal WM and of considering regions beyond the “usual suspects”.

## Highlights

• Digit span forward and backward performance has unique intrinsic neural correlates.
• Dorsal anterior cingulate gyrus plays distinctive roles in forward and backward span.
• Angular gyrus and subcallosum associated with forward digit span performance depending on age.
• Visual cortex and ventrolateral PFC linked to backward digit span performance depending on age.
• Age-related brain–behavior relationship changes are more robust for forward span.

## 1 Introduction

Working memory is the ability to maintain and manipulate information online during goal-directed task performance. This ability is central to the acquisition of knowledge and skills (e.g., reading, numerical calculation, and problem solving) throughout development and predicts academic achievement (Alloway and Alloway, 2010, Hitch et al., 2001). Verbal WM is particularly important given the role of linguistic processes in high-order cognitive functions. Behaviorally, the ability to hold information in memory (maintenance) increases during early childhood, while the ability to operate and use the stored information (manipulation) improves most dramatically during late childhood and adolescence (Gathercole, 1999). The development in brain architecture underlying these increases in WM capacity has yet to be determined.

Task-based activation studies of WM development highlighted shifts from diffuse to focal patterns of activation, and increased recruitment of brain areas implicated in WM for adults (Bunge and Wright, 2007). Studies emphasize the frontoparietal network and its maturational status as major determinants of WM performance (Klingberg, 2006, Sander et al., 2012). Efforts to understand the development of WM components (maintenance, manipulation) have focused on prefrontal cortex (PFC) and superior parietal lobe (SPL) (Crone et al., 2006), attributing the late development of manipulation ability to protracted maturation of dorsolateral PFC (DLPFC) and SPL (Casey et al., 2008, Diamond, 2002). This differs from ventrolateral PFC (VLPFC), which is commonly implicated in both maintenance and manipulation (Owen et al., 2000), and matures during early childhood (Diamond, 2002). However, few studies provide insights into how changes in functional interactions between regions may contribute to WM development.

Task-based connectivity studies of verbal WM emphasized maturational changes of within and between network functional interactions. For example, van den Bosch et al. (2014) reported age-related functional connectivity changes in children and adolescents within frontoparietal, motor and cingulate networks. Using a similar task, Finn et al. (2010) found longitudinal decreases in connectivity between hippocampus and lateral PFC with age. Combined, these results emphasize the need to consider a broad range of areas and their functional interaction, rather than any single system.

A central challenge for task-based functional magnetic resonance imaging (fMRI) is designing tasks that equivalently probe WM in different age groups (Church et al., 2010, Luna et al., 2010). Previous studies addressed this challenge by either statistically controlling for performance (van den Bosch et al., 2014) or matching performance, i.e., selecting low performers from adults to match the performance of children (Crone et al., 2006). However, statistically controlling for performance may hinder the ability to detect age effects (van den Bosch et al., 2014), as performance typically depends on age (see Satterthwaite et al., 2013b for an example of examining the unique effects of age and performance). Also, adults with low performance may not be appropriately representative.

Not limited by tasks, resting-state fMRI (R-fMRI) is a powerful tool for mapping maturational changes in brain functional organization (Dosenbach et al., 2010) and indexing inter-individual differences in cognition and behavior (Kelly et al., 2008, Koyama et al., 2011). Most R-fMRI studies of verbal WM have focused on adults (Gordon et al., 2014, Jolles et al., 2013, Takeuchi and Kawashima, 2012). One study in children examined the maturation of functional connectivity underlying the improvement of cognitive control, a core component of manipulation (Barber et al., 2013). Those authors found that the intrinsic anticorrelation between the task positive and default network was greater in adults than children. In addition, the strength of this anticorrelation was associated with inhibitory control performance across groups. These results suggested that the development of this anticorrelation supports mature inhibitory control. However, this pioneering effort had a number of limitations: reliance on a seed-based correlation approach, treating age as a categorical variable, and lack of an adolescent group.

Using R-fMRI, we systematically examined neural indices of WM performance in a cross-sectional developing sample (ages: 7–17 yrs) utilizing a broad range of data-driven approaches. Compared to traditional seed-based correlation analysis, data-driven approaches do not require a priori hypotheses and allow for identifying previously overlooked brain–behavior relationships. Using a relatively large sample (*n* = 68), we treated age as a continuous variable. WM was assessed using the Wechsler Intelligence Scale for Children (WISC-IV; Wechsler, 2004) Digit Span (DS) subtest, which includes: DS Forward (DSF) and DS Backward (DSB). These tasks were selected because they are well-validated measures commonly used in educational and clinical evaluation (Gathercole and Alloway, 2006), which increases the ecological validity of our findings. The available age-normalized scores also allow comparisons of performance across different ages.

The cognitive literature has suggested that DSF and DSB rely on shared and distinct WM components (Hale et al., 2002, Reynolds, 1997). DSF is thought to depend on the ability to maintain information in the “phonological loop” and is strongly associated with language development (Baddeley, 2012). DSB has additional executive control requirements to transform and manipulate information (e.g., reverse the digit sequence). Thus, DSB is more reflective of cognitive control (Baddeley, 2012) and involves visual-spatial skills (St Clair-Thompson and Allen, 2013). Because the DS total (DST = DSF + DSB) score is widely used to index verbal WM abilities (Walshaw et al., 2010), we examined the aggregate, as well as distinct intrinsic brain correlates of DSF and DSB via two regression analyses: for aggregate analyses, we included DST as the variable of interest in a model; for distinct analyses, we included DSF and DSB scores in the same model to control the effect of the other.

R-fMRI analyses included two types of data-driven approaches: (1) a set of commonly used regional derivatives that are amenable to univariate voxel-wise analysis, including: Degree Centrality (DC; Zuo et al., 2012), Regional Homogeneity (ReHo; Zang et al., 2004), fractional Amplitude of Low-Frequency Fluctuations (fALFF; Zou et al., 2008); and Voxel-Mirrored Homotopic Connectivity (VMHC; Zuo et al., 2010); and (2) a multivariate analytic framework: Multivariate Distance Matrix Regression (MDMR: Shehzad et al., 2014). MDMR identifies voxels whose whole-brain connectivity patterns vary significantly with verbal WM performance, age, or their interactions and provide a more comprehensive characterization of brain–behavior relationships. See Table 1 for definition/interpretation of each approach. Given continued controversies regarding optimal R-fMRI preprocessing strategies (Power et al., 2014, Yan et al., 2013c), we also evaluated the robustness of our results to preprocessing decisions.

**Table 1:** Definition and interpretation of each approach.

Approaches	Calculation	Interpretation
DC	For a binary graph, DC is the number of edges (i.e. significant functional connection) connecting to a given node (i.e. a voxel) (Zuo et al., 2012)	Measures the relative importance of an area in the brain's functional connectivity graph. Inter-individual differences in DC may indicate variation in the number of connections with a brain area, which may reflect variation in the biological processes that subtend local and long-range functional connectivity
ReHo	The Kendall's coefficient of concordance of the time series of a given voxel with those of its 26 nearest neighboring voxels (Zang et al., 2004)	Measures local synchronicity that reflects the functional homogeneity of brain areas (Jiang et al., 2014). Differences in ReHo across participants may indicate variation in the biological processes that subtend local functional connectivity
fALFF	ALFF is the standard deviation of the bandpass filtered (0.01–0.1 Hz) fMRI signal of a given voxel. fALFF is the ratio of ALFF to the average amplitudes of the entire frequency range (Zou et al., 2008)	Measures power of a brain area's spontaneous activity that falls within the frequencies typically associated with resting state functional connectivity
VMHC	Pearson's correlation coefficient between the time series of a given voxel and that of its symmetrical interhemispheric counterpart (Zuo et al., 2010)	Measures the strength of interhemispheric connectivity, which is thought to reflect hemispheric functional specialization (Stark et al., 2008)
MDMR	The computation of this multivariate approach included three steps (Shehzad et al., 2014). For details, see Section 2.4.3	Identifies voxels whose whole-brain connectivity patterns vary significantly with a phenotypic variable, which takes all functional interactions within the brain into consideration simultaneously

Note: DC, degree centrality; ReHo, regional homogeneity; fALFF, fractional amplitude of low frequency fluctuations; VMHC, voxel mirrored homotopic connectivity; MDMR, multivariate distance matrix regression; ALFF, amplitude of low frequency fluctuations.

## 2 Materials and methods

### 2.1 Participants

Seventy-two right-handed typically developing children and adolescents (age range: 7.13–16.84 yrs; mean = 12.13 ± 2.74 yrs, 40 males, intelligence quotient [IQ] > 80) were included from studies conducted at the Child Study Center at the New York University (NYU) Langone Medical Center between 2009 and 2013. None of the participants had chronic medical conditions, DSM-IV-TR Axis-I psychiatric diagnoses (based on Schedule of Affective Disorders and Schizophrenia for Children-Present and Lifetime Version: (K-SADS-PL; Kaufman et al., 1996), symptoms of ADHD (ADHD index of the Conners Parent Rating Scale-Revised-Long Version < 65) (Conners, 1997), or other behavioral or emotional problems (Child Behavioral Checklist Total Problems score < 63) (Achenback and Rescorla, 2001). Outlier analyses were performed on DS performance and in-scanner head motion. No participant was excluded due to DS performance. Within scanner head movement was quantified using mean frame-wise displacement (FD) (Power et al., 2012). Four participants were excluded due to either mean FD above three times inter-quartile-range or FD exceeding 0.2 mm in >50% of the volumes.

The final sample (*n* = 68) was evenly distributed across the age range (Supplementary Fig. 1). The younger and older participants (top and bottom tercile sorted by age: *n* = 23 per group) did not significantly differ in sex, socioeconomic status (Hollingshead Index of Social Position, Hollingshead, 1975), full-scale IQ (Wechsler Abbreviated Scale of Intelligence; Wechsler, 1999), or head motion quantified using mean FD (younger: 0.12 ± 0.05 mm; older: 0.11 ± 0.05; *p* > 0.20). The older group was more strongly right-handed than the younger group (*t*_(44)_ = 2.48, *p* = 0.017) (Edinburgh Handedness Inventory, Oldfield, 1971). This factor was controlled in group analyses. The study was approved by the institutional review boards of NYU and NYU Langone Medical Center. Prior to participation, written assent and consent were obtained from children and their parents/legal guardians, respectively.

### 2.2 Digit span tests

We employed the DS tests from the WISC-IV (Wechsler, 2004). To ensure uniformity of administration, the stimuli were presented via a computer generated female voice (Mac OS X ‘say’ command). The DSF task measures an individual's ability to encode and maintain sequentially presented auditory-verbal information by having participants repeat the sequence aloud in the same order as presented immediately after hearing the sequence. The DSB task additionally measures an individual's ability to manipulate information in WM by repeating the numbers in reverse order. For each task, there are eight levels of difficulty (two trials/difficulty level), which differ with respect to sequence length (i.e., 2–9 numbers). Participants started with the easiest level, and only advanced to the next level if at least one of the two trials was correct. Age-normalized scores were generated for DSF, DSB, and DST scores separately per the WISC-IV manual.

### 2.3 MRI data acquisition

We acquired imaging data using a Siemens Allegra 3.0 Tesla scanner (Siemens, Iselin, NJ, USA), located at the NYU Center for Brain Imaging. Each participant completed a 6-min resting scan, which was comprised of 180 contiguous whole-brain functional volumes acquired using a multi-echo echo-planar imaging (EPI) sequence (effective TE = 30 ms; TR = 2000 ms; flip angle = 90°; 33 slices; voxel-size = 3 mm × 3 mm × 4 mm; Field of View [FOV] = 240 mm × 192 mm). During the scan, 58 participants rested with their eyes open and 10 with their eyes closed. Proportions of scans with eyes open/closed did not differ significantly (*p* > 0.20) between the younger tercile (18/5) and the older tercile (20/3). A high-resolution T1-weighted anatomical image was also acquired using a magnetization prepared gradient echo sequence (MPRAGE, TR = 2530 ms; TE = 3.25 ms; TI = 1100 ms; flip angle = 7°; 128 slices; FOV = 256 mm; acquisition voxel size = 1.3 mm × 1.3 mm × 1.3 mm).

### 2.4 Primary analyses

#### 2.4.1 Imaging preprocessing

Imaging data were preprocessed using an Alpha version of the Configurable Pipeline for the Analysis of Connectomes (CPAC 0.3.3, http://fcp-indi.github.io/docs/user/index.html). For each participant, the first five volumes were removed to allow the signal to reach T1 equilibrium, leaving a total of 175 volumes for final analysis. Image preprocessing steps included: slice timing correction, realignment to the mean EPI image to correct for motion, grand mean-based intensity normalization (all volumes scaled by a factor of 10,000), nuisance regression, spatial normalization, temporal band-pass filtering (0.01–0.1 Hz, except for fALFF), and spatial smoothing.

Nuisance regression was performed to remove nuisance variation due to physiological processes (e.g., respiration and cardiac processes) and motion. The model included linear and quadratic trends, mean signals from white matter, mean signals from cerebrospinal fluid (CSF), and the Friston-24 motion parameters (6 head motion, their values from one time point before, and the 12 corresponding squared items) (Friston et al., 1996). To further account for residual systematic variation not accounted by these regressors, we applied mean regression (MR) strategy to normalize the data by including the global mean of a given derivative as a nuisance regressor in group analyses (Yan et al., 2013c). Compared to other normalization approaches (e.g., global signal regression: GSR), MR avoids introducing artifactual relationships with the global mean.

Depending on the approach (e.g., DC, ReHo, MDMR), spatial normalization and spatial smoothing happened either before or after the derivative was calculated see Sections 2.4.2 and 2.4.3 for details. Spatial normalization included: (1) structural-to-standard registration using Advanced Normalization Tools (ANTs; Avants et al., 2011), which has been demonstrated to have superior performance compared to other commonly used registration algorithms (Klein et al., 2009, Klein et al., 2010); (2) functional-to-structural registration using FLIRT with a 6-degrees of freedom linear transformation. This co-registration was further refined using Boundary-based Registration implemented in FSL (Greve and Fischl, 2009); and (3) functional-to-standard registration using ANTs via applying the transformation matrices obtained from the previous two steps. All univariate approaches were warped to 2 mm^3^ MNI space. In our experience, 2 mm^3^ resolution tends to be slightly more optimal than 3 mm^3^ in capturing subtle anatomical variations across participants; though differences in findings across resolutions are relatively small. For MDMR-based analyses, we made an exception and used 3 mm^3^ voxel size to balance our desire for spatial precision with the realities of the computational complexity of the multivariate approach. Prior work by our lab has reported high concordance in MDMR results across different resolutions (Shehzad et al., 2014). Spatial smoothing was performed using a Gaussian kernel (FWHM = 6 mm).

#### 2.4.2 Univariate voxel-wise approaches

For each participant, we computed the following four derivatives based on the R-fMRI data (DC was calculated in standard space and then smoothed. ReHo and fALFF were calculated in EPI space and then registered to MNI space and smoothed. VMHC was calculated on smoothed data in symmetrical template in standard space):

(1) DC identifies the most connected nodes within the whole-brain functional network (Zuo et al., 2012). To calculate voxel-wise DC, a study-specific group mask was first created to include voxels (in MNI space) present in at least 90% of participants. Voxel-based graphs were then generated within this mask in standard space: each voxel (2 mm^3^) constitutes a node, and each functional connection (i.e., Pearson correlation) between a pair of voxels is an edge. This graph was then represented by a binary undirected adjacency matrix obtained via thresholding each correlation at *r* > 0.25 (Buckner et al., 2009), here equivalent to *p* < 0.0008. DC was calculated by counting the number of significant correlations between a given voxel and all other voxels.
(2) ReHo measures local coherence of intrinsic brain activities, defined as the Kendall's coefficient of concordance of the time series of a given voxel with those of its 26 nearest neighboring voxels (Zang et al., 2004).
(3) fALFF measures the intensity of intrinsic brain activity, defined as the ratio of power within the low frequency range (0.01–0.1 Hz) to the power of the entire frequency band (Zou et al., 2008).
(4) VMHC measures inter-hemispheric functional connectivity, defined as the Fisher's Z transformed Pearson's correlation coefficient between the time series of a given voxel and that of its symmetrical inter-hemispheric counterpart (Zuo et al., 2010).

Group analyses were performed using general linear models (GLM) implemented in a toolbox for Data Processing & Analysis of Brain Imaging (DPABI; Yan and Zang, 2010). The following two regression models were constructed to examine the aggregated and distinct neural correlates of DSF and DSB and the age-related changes in brain–behavior relationships:

• A given derivative = b0 + b1 × DST + b2 × (DST × Age) + b3 × Age + b4 × FIQ + b5 × Handedness + b6 × meanFD + error
• A given derivative = b0 + b1 × DSF + b2 × DSB + b3 × DSF × Age + b4 × DSB × Age + b5 × Age + b6 × FIQ + b7 × Handedness + b8 × meanFD + error

For DST, DSF, and DSB, age-normalized scores were included as regressors of interest. Age, handedness, and full-scale IQ were included as nuisance covariates. IQ was included because general intelligence and WM were correlated, as expected (DSF: *r* = 0.39, *p* = 0.001; DSB: *r* = 0.35, *p* = 0.004). Mean FD was also included to control for the residual effect of head motion. We opted to employ group-level corrections for motion over scrubbing (Power et al., 2014), as recent work suggested that scrubbing offers little advantage over group-level corrections, is less conservative when motion correlates with a between-subject variable of interest, and can correct incompletely (Satterthwaite et al., 2013a, Yan et al., 2013a). Because some covariates correlated with each other (e.g., DSF, DSB, and IQ), we performed Belsley collinearity diagnostics confirming that our regression models were not multicollinear. Group analyses were constrained within the study-specific mask used to calculate DC. Results were corrected for multiple comparisons using Gaussian Random Field theory (GRF: voxel threshold: *Z* > 2.33, cluster-level threshold: *p* < 0.05). Although the four univariate measures are believed to assess distinctive aspects of intrinsic brain function (Aiello et al., 2015, Zuo and Xing, 2014), they are not entirely independent of one another – making correction for the number of measures (i.e., four) overly conservative. Thus, we opted not to correct for the number of measures. To address any possible concerns about this decision, we did repeat our analyses with Bonferroni correction for the number of univariate measures, finding the vast majority of findings remained (Supplementary Fig. 5).

To understand the nature of the DS × Age interaction, we fit the DS scores (DST, DSF, or DSB), age, and the beta values obtained from group analyses to the following model to compute the predicted derivative score: A predicted derivative = b1 × DS + b2 × Age + b3 × (DS × Age). The original DS and age scores were linearly spaced with 100 points between minimal and maximum values to obtain a finer grid. A 100 × 100 grid was obtained for the DS × Age interaction by multiplying spaced DS and age scores. Region-of-interest (ROI) mean beta values were extracted for a given cluster based on the corresponding effect (b1: main effect of DS; b2: main effect of Age; b3: DS × Age). Predicted values were then plotted as a function of DS performance and age to show how brain–behavior relationships differ in relation to age.

#### 2.4.3 Multivariate distance matrix regression approach

The MDMR-based approach was carried out using the R package connectir (http://czarrar.github.io/connectir) on spatially normalized and smoothed data (Shehzad et al., 2014). The computation was constrained on a study-specific group mask including only voxels (in 3 mm^3^ MNI space) present in all participants and in the MNI152 25% gray-matter probability mask provided by FSL.

The detailed procedure of MDMR analysis can be found in Shehzad et al. (2014). Briefly, for a given voxel, MDMR analysis included three steps: (1) calculate the Pearson's correlation between the time series of a given voxel and that of all voxels within the group mask to assess the whole-brain functional connectivity of this voxel; (2) calculate the distance between the whole-brain connectivity patterns for every possible pairing of participants. This calculation resulted in an *n* × *n* distance matrix where *n* is the number of participants; and (3) use a pseudo-F statistic (MDMR; Zapala and Schork, 2012) to test the extent to which DS performance and DS × Age interactions explain the distances of whole-brain functional connectivity observed between participants in step 2. The regressors used in MDMR were the same as the ones used in the univariate voxel-wise analysis. The significance of the pseudo-*F* statistic is assessed using a permutation test (15,000 permutations). Steps 1–3 were repeated for every voxel within the group mask. Multiple comparisons were corrected using GRF (voxel-level: *Z* > 1.65 which corresponds to *p* < 0.05 in an *F*-test; cluster-level: *p* < 0.05).

The MDMR approach can inform the presence of a relationship between DS performance and whole-brain connectivity patterns for specific bran areas, but does not specify the nature of the association (i.e., the specific connections involved and their directions). To further characterize the MDMR results, we performed follow-up ROI-based intrinsic functional connectivity (iFC) analyses using MDMR detected regions as seed ROIs. The voxels exhibiting a significant main effect of DS or DS × Age interaction were split into clusters using a nearest neighbor algorithm. For each seed ROI, the average time-series across all voxels within the ROI were extracted and correlated with all voxels within the group mask using Pearson's correlation. Correlation values were transformed to Fisher's *Z* scores to provide a whole-brain connectivity map. The same group analyses as used for MDMR were subsequently performed to identify brain areas whose iFC with the seed ROI are significantly associated with DS performance or DS × Age interaction. Results were corrected for multiple comparisons using GRF (*Z* > 2.33; *p* < 0.05).

### 2.5 Secondary analyses

Given concerns regarding artifactual findings induced by nuisance signal correction strategies, we further evaluated the robustness of our findings to various commonly used preprocessing strategies. For univariate approaches, besides the strategy used in the primary analysis (MR: including white matter and CSF at the individual-level and global mean of a given derivative at the group-level), we repeated the analysis using four other nuisance correction strategies: (1) component-based noise correction methods (CompCor): including signals of five principal components derived from “noise ROIs” (e.g., white matter and CSF) at the individual level (Behzadi et al., 2007); (2) GSR: including signals from white-matter, CSF, and global signal at the individual level (Fox et al., 2009); (3) global correlation (GCor): including white matter and CSF at the individual level and GCor (i.e., average correlations between all possible pairs of voxels within the brain) at the group level to correct for global variations in connectivity (Saad et al., 2013); and (4) the basic model: including white matter and CSF at the individual level. For all these strategies, linear trends, quadratic trends, and Friston-24 motion parameters were also included in the models. For MDMR, the basic model was applied in the primary analysis because mean regress does not apply. Additionally, Strategies 1, 2, and 3 were also tested.

## 3 Results

### 3.1 Behavioral results

**Fig. 1:**
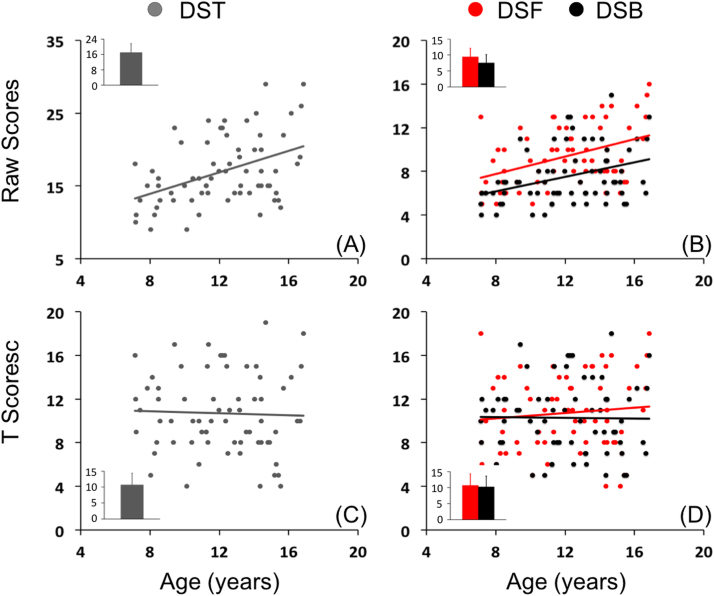
Behavioral results of digit span task. Raw scores (A, B) and *T* scores (C, D) of digit span total (DST: grey), DS forward (DSF: red), and DS backward (DSB: black) are plotted as a function of age. The mean and standard deviation of each score type are plotted in the inserted bar graph.

Consistent with prior work (Gathercole et al., 2004), DS raw scores were significantly correlated with age (Fig. 1; forward: *r* = 0.41, *n* = 68 unless otherwise stated, *p* = 0.001; backward: *r* = 0.35, *p* = 0.003; total: *r* = 0.43, *p* = 0.0003). The average backward raw scores (7.54 ± 2.57) were significantly lower than forward raw scores (9.41 ± 2.69) (*t* = 6.66, *p* < 0.0001), confirming that the backward task is more difficult. As expected, age-normalized T scores for total, forward, and backward DS were not significantly correlated with age (*p* > 0.20). Standard forward (10.74 ± 3.52) and backward (10.28 ± 3.31) scores were highly correlated (*r* = 0.53, *p* < 0.001). Two-sample *t*-tests were performed on DST, DSF, and DSB standard scores of younger versus older participants (top and bottom age tercile) to confirm that these two groups did not differ in behavioral indices (*p* > 0.20).

### 3.2 Imaging results

#### 3.2.1 Primary analyses: Main effect of digit span performance

**Fig. 2:**
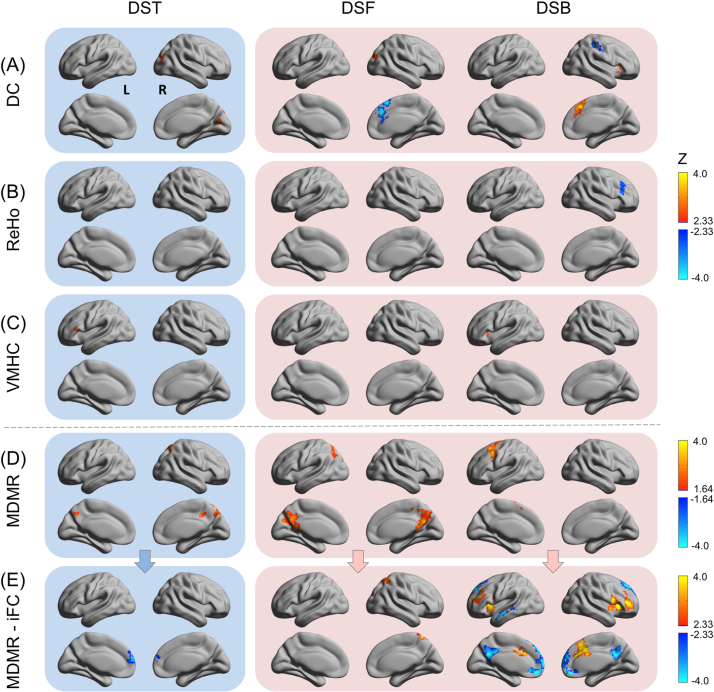
Main effect of digit span. For each approach, the main effect of digit span total (DST) was tested in one model (light blue shaded) and the main effect of DS forward (DSF) and DS backward (DSB) were tested together in another model (pink shaded). Panel A–C: univariate approaches, including Degree Centrality (DC), Regional Homogeneity (ReHo), and Voxel-Mirrored Homotopic Connectivity (VMHC); no significant results were observed for fractional Amplitude of Low-Frequency Fluctuations (fALFF). Panel D: multivariate approach, Multivariate Distance Matrix Regression (MDMR). *Z* scores for ROIs exhibiting significant main effects are plotted on lateral and medial view of a surface map in MNI space using BrainNet Viewer (http://www.nitrc.org/projects/bnv/) (L = left; R = right). For univariate approaches, warm colors indicate that greater values of a derivative are associated with better DS performance (positive relationship) and cold colors indicate that greater values of a derivative score are associated with worse DS performance (negative relationship). Clusters identified by MDMR (Panel D: one cluster was identified for each effect) were followed up by an intrinsic functional connectivity (MDMR-iFC) analysis (Panel E) to understand how specific connections drive the relationship between DS performance and the seed brain regions’ whole-brain connectivity patterns. (For interpretation of the references to color in this figure legend, the reader is referred to the web version of the article.)

The brain areas related to overall DS performance were identified by the main effect of DST (Fig. 2 DST, Table 2). Overall, each R-fMRI derivative revealed a distinctive set of associations with little overlap. Specifically, greater DC within the right lateral and medial visual area, and greater VMHC within the pars triangularis of the inferior frontal gyrus were associated with better performance. The MDMR approach identified a cluster composed of portions of bilateral precuneus extending into the right lateral visual area whose whole-brain connectivity patterns significantly vary with DST score. This cluster overlapped with portions of fronto-parietal and dorsal attention networks as defined by Yeo et al. (2011). Using this cluster as a seed, follow-up iFC analysis revealed that connectivity between this seed ROI and the anterior core regions of default network (including medial prefrontal cortex/anterior cingulate cortex: MPFC/ACC) were negatively correlated with overall performance (Fig. 2E DST, Supplementary Table 1).

**Table 2:** Brain areas associated with verbal working memory performance regardless of age: main effect of digit span.

Approaches	Main effect	Region (Harvard-Oxford atlas)	BA	Network (Yeo et al., 2011)	Center of mass (MNI)			Volume (# voxels)
					*X*	*Y*	*Z*	
DC	DST	R LOC, R intracalcarine cortex/occipital pole	17/18/19	Visual	21	−81	23	894
	DSF	R SFG/ACC/paracingulate gyrus	8/24/32	Control	7	30	38	1010
		R LOC/occipital pole	19/39	Visual	25	−82	27	468
	DSB	R pre- and post-central gyrus	2/3/4/40	SomMot	40	−28	54	631
		R frontal operculum/orbital cortex, R insula	47	VentAttn	36	24	1	443
		R SFG/paracingulate gyrus	8/32	Control	6	28	43	716
ReHo	DSB	R MFG	44/45/46	Control	41	28	31	655
VMHC	DST	IFG, pars triangularis	45	Default	46	24	10	148
	DSB	Frontal operculum/orbital, insula	47	VentAttn	33	23	−1	316
MDMR	DST	B precuneus, R LOC	7	DorsAttn/Control	16	−61	47	388
	DSF	B precuneus, L LOC	7/23	Default/Control/DorsAtt/Visual	−5	−61	32	1289
	DSB	L SFG/MFG	6/8/9	Control/Default/DorsAttn	−27	9	51	348

Note: DC, degree centrality; ReHo, regional homogeneity; VMHC, voxel mirrored homotopic connectivity; MDMR, multivariate distance matrix regression; DST, digit span total; DSF, digit span forward; DSB, digit span backward; R, right; LOC, lateral occipital cortex; SFG, superior frontal gyrus; ACC, anterior cingulate cortex; MFG, middle frontal gyrus; IFG, inferior frontal gyrus; B, bilateral; L, left; BA, Brodmann area; Control, frontoparietal control network; SomMot, somatomotor network; VentAttn, ventral attention network; DorsAttn, dorsal attention network. Voxel size for univariate approaches is 2 mm × 2 mm × 2 mm and for multivariate approach is 3 mm × 3 mm × 3 mm.

The brain areas uniquely related to DSF and DSB performance were identified from the main effect of DSF and DSB scores included in the same model (Fig. 2 DSF and DSB, Table 2). As DST is composed of DSF and DSB, we wondered whether its neural correlates reflect this additive relationship. We found that part of the unique effect of DSF is reflected in DST (i.e., DC within the lateral visual area and MDMR within the precuneus), but none of the unique effect of DSB was captured by DST, suggesting combining diluted these unique effects (Fig. 2 compare DST with DSF and DSB).

Adjusting for DSB, DSF was uniquely associated with DC within the right lateral visual cortex and the whole-brain connectivity within a cluster composed of bilateral precuneus/posterior cingulate cortex (PCC) extending into the left lateral visual area. MDMR-guided iFC analysis revealed that the connectivity between this cluster and the motor subdivision of precuneus extending into the right lateral visual area was significantly positively correlated with DSF performance. Interestingly, DC within the dorsal ACC area was commonly associated with DSF and DSB performance but in opposite directions (i.e., negative for DSF and positive for DSB).

**Fig. 3:**
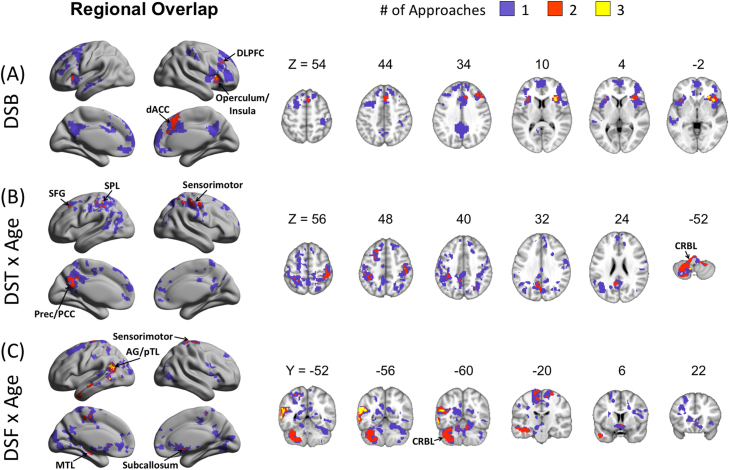
Regional overlap between approaches. Surface and slice maps are shown to depict the extent of overlap between approaches for the main effect of digit span backward (DSB, Panel A), DS total (DST) × Age interaction (Panel B), and DS forward (DSF) × Age interaction (Panel C). For the main effect of DST and DSF, as well as the DSB × Age, no regional overlap was observed between approaches. Regions overlapping across either two to three approaches are represented by red and yellow, respectively. Regions only detected by one approach are represented in purple. Locations of the axial (*Z*) and coronal (*Y*) slices are indicated in MNI coordinates. DLPFC: dorsolateral prefrontal cortex; dACC: dorsal anterior cingulate gyrus; SFG: superior frontal gyrus; SPL: superior parietal lobe; Prec/PCC: precuneus/posterior cingulate gyrus; CRBL: cerebellum; AG/pTL; angular gyrus/posterior temporal lobe; MTL: medial temporal lobe.

As expected, the more demanding DSB task was uniquely associated with several key nodes of task-positive networks, including frontal opercular and anterior insular cortex (portions of ventral attention network). These regions were highlighted by three approaches: DC, VMHC, and MDMR-guided iFC analyses (Fig. 3A). Better DSB performance was associated with greater DC and VMHC within this cluster, and with greater connectivity between this cluster and a cluster composed of the left frontal eye fields (FEF)/premotor area which was identified by MDMR. Using FEF/premotor area as a seed, iFC analysis revealed that connectivity between this seed and other nodes with task-positive networks (e.g., the DLPFC, VLPFC, ACC, frontal operculum, and anterior insula) were positively correlated with DSB performance. In contrast, the connectivity between this seed and areas corresponding to the default network (i.e., MPFC/ACC, PCC/precuneus, and lateral temporal lobe) was negatively associated with DSB score. Areas commonly detected by two approaches include the right DLPFC (in ReHo and MDMR-guided iFC analysis) and the right dorsal ACC (dACC, in DC and MDMR-guided iFC analysis). Finally, areas identified by a single approach included the right sensorimotor area (in DC).

#### 3.2.2 Primary analyses: Interaction effect between digit span performance and age

**Fig. 4:**
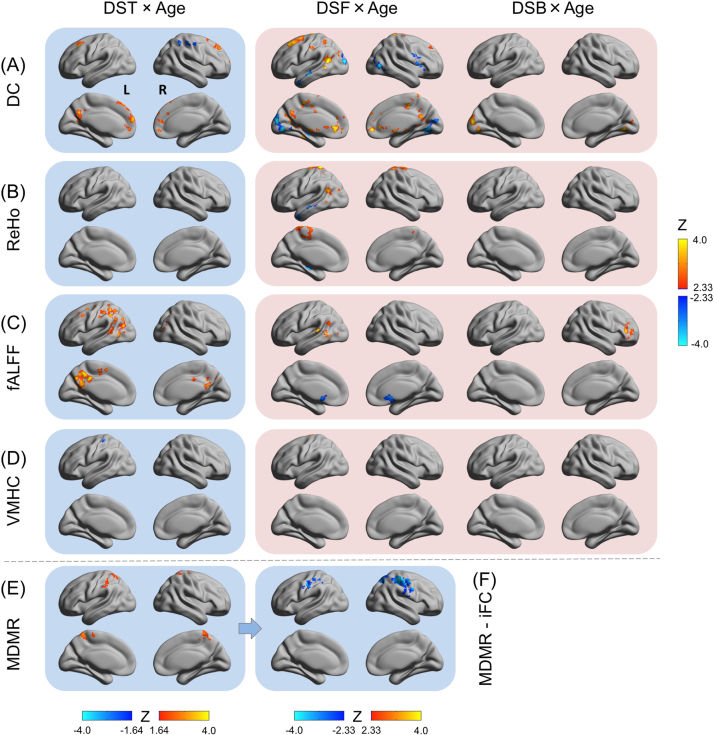
Interaction between digit span and age. For each approach, DS total (DST) × Age was tested in one model (light blue shaded) and DS forward (DSF) × Age and DS backward (DSB) × Age were tested together in another model (pink shaded). Panels A–D: univariate approaches, including Degree Centrality (DC), Regional Homogeneity (ReHo), fractional Amplitude of Low-Frequency Fluctuations (fALFF), and Voxel-Mirrored Homotopic Connectivity (VMHC). Panel E: multivariate approach, Multivariate Distance Matrix Regression (MDMR). The *Z* scores for ROIs exhibiting significant interaction effects are plotted onto an MNI space surface map (L = left; R = right). The pattern of positive (warm color) and negative (cold color) interactions is illustrated in Fig. 5 using several exemplar clusters. Note that MDMR detected a significant interaction with age only for DST. Thus, the MDMR-guided iFC intrinsic functional connectivity (MDMR-iFC) analysis was only performed for DST × Age (Panel F). (For interpretation of the references to color in this figure legend, the reader is referred to the web version of the article.)

The brain–behavior relationships that are modulated by age were detected by the DS × Age interaction (Fig. 4, Table 3). Several clusters were detected by at least two approaches yielding a significant DST × Age interaction (Fig. 3B), including the right pre- and postcentral gyrus and superior parietal lobe (SPL, in DC and MDMR-guided iFC analysis), the left postcentral gyrus/SPL (in fALFF and MDMR-guided iFC analysis), the left superior frontal gyrus (SFG, in DC and fALFF), the left PCC/precuneus (in DC and fALFF), and cerebellum (in DC and ReHo). Areas detected by a single approach included: bilateral MPFC/ACC in DC; left SPL, left temporoparietal junction, and bilateral lateral occipital cortex in fALFF.

**Table 3:** Age-dependent brain–behavior associations: digit span by age interaction.

Approaches	Effect	Region (Harvard-Oxford atlas)	BA	Network	Center of mass (MNI)			Volume (# voxels)
					*X*	*Y*	*Z*	
DC	DST × Age	R postcentral gyrus/SPL	2/3	DorsAttn	41	−36	53	494
		L cerebellum	–	–	−24	−59	−52	668
		L PCC/precuneus	17/23	Default	−9	−58	27	495
		L SFG/MFG	8/9	Default	−20	22	48	782
		B SFG/B frontal pole/B paracingulate gyrus ACC	9/10/32	Default	7	42	25	1806
	DSF × Age	L temporal pole/MTG, L hippocampus	20/21	Default	−47	−11	−20	781
		R frontal operculum cortex/IFG/precentral gyrus	6	DorsAttn/VentAttn	39	8	21	893
		L occipital pole, B lingual gyrus, B cerebellum	18/19/37	VentAttn/Control/Visual	−4	−69	−18	7872
		R PCC/precuneus	17/23/30	Visual/Default	11	−49	9	753
		L SFG	6/8	Default/DorsAttn	−20	12	56	986
		L parahippocampal gyrus/thalamus	30/35	Limbic	−12	−18	−7	1043
		B pre- and postcentral gyrus/SPL	4	SomMot/DorsAttn	−14	−27	45	2336
		L AG/LOC, L precuneus	17/19/37/39	Visual/DorsAttn/Default	−32	−60	19	2396
		B frontal pole/paracingulate gyrus	10/11/32	Default/Control	−8	36	11	2840
	DSB × Age	B lingual gyrus, L occipital pole	17/18	Visual	1	−79	2	1429
ReHo	DST × Age	B cerebellum	–	–	−19	−53	−47	1793
	DSF × Age	L MTG/temporal pole, L hippocampus	20/21	Default	−47	−13	−20	821
		L fusiform gyrus, L cerebellum	37	Control/VentAttn	−32	−57	−40	1294
		L MTG/AG/LOC	21/22/37/39	DorsAttn/Default	−49	−61	15	935
		B pre- and postcentral gyrus	4/6	SomMot	−1	−29	66	2620
fALFF	DST × Age	B brain stem, B cerebellum	–	Limbic	−2	−37	−48	849
		R LOC	7/19/37/39	DorsAttn	37	−65	28	797
		L SFG/MFG, L precentral gyrus, L juxtapositional lobule cortex	6	DorsAttn/SomMot	−19	−2	52	928
		L SPL/SMG/AG, L postcentral gyrus, B Precuneus/PCC, L LOC	23/39/40	Default/Control/DorsAttn	−27	−52	31	5458
	DSF × Age	B subcallosal cortex/accumbens	11/25	Limbic	4	14	−9	735
		L STG/MTG/AG/LOC	21/22/37/39	DorsalAttn/Default	−56	−55	10	1044
	DSB × Age	R frontal pole	45/46	Control	38	38	13	777
VMHC	DST × Age	Postcentral gyrus	3/4	SomMot	38	−30	64	199
	DSF × Age	Cerebellum	–	–	−10	−68	−19	162
		Precentral gyrus	4	SomMot	−12	−17	66	157
MDMR	DST × Age	B SPL/B postcentral gyrus/B precuneus	5/40	SomMot/DorsAttn	−8	−42	58	552

Note: DC, degree centrality; ReHo, regional homogeneity; fALFF, fractional amplitude of low frequency fluctuations; VMHC, voxel mirrored homotopic connectivity; MDMR, multivariate distance matrix regression; DST, digit span total score; DSF, digit span forward; DSB, digit span backward; R, right; SPL, superior parietal lobule; L, left; PCC, posterior cingulate cortex; SFG, superior frontal gyrus; MFG, middle frontal gyrus; B, bilateral; ACC, anterior cingulate gyrus; MTG, middle temporal gyrus; IFG, inferior frontal gyrus; AG, angular gyrus; LOC, lateral occipital cortex; SMG, supramarginal gyrus; STG, superior temporal gyrus; BA, Brodmann Area; DorsAttn, dorsal attention network; VentAttn, ventral attention network; Control, frontoparietal control network; SomMot, somatomotor network. Voxel size for univariate approaches is 2 mm × 2 mm × 2 mm and for multivariate approach is 3 mm × 3 mm × 3 mm.

Because DSF × Age and DSB × Age were examined in the same model, each could reveal age-dependent brain–behavior relationships uniquely for each task. We first compared DSF × Age and DSB × Age with DST × Age to examine whether DST interaction reflect the aggregate effect of both tasks. Similar to the main effect, part of the DSF × Age interaction was captured by DST × Age (DC within the medial core areas of the default network, and fALFF within the angular gyrus/posterior temporal lobe [AG/pTL]), but no unique interaction for DSB was observed in DST (Fig. 4, compare DSF × Age and DSB × Age with DST × Age).

For the unique DSF × Age interaction, a cluster around AG/pTL was convergently identified by three approaches (DC, ReHo, and fALFF: Fig. 3C). Clusters identified by two approaches included bilateral sensorimotor cortex (DC and ReHo), left lateral and medial temporal cortex (DC and ReHo), bilateral cerebellum (DC and ReHo, or DC and VMHC), and bilateral subcallosum (DC and fALFF). Areas detected by one approach (DC) include: bilateral ACC/MPFC, left SFG, bilateral PCC/precuneus, left parahippocampal gyrus and thalamus, the right frontal operculum/precentral gyrus, and bilateral cuneus and lingual gyrus. Compared to the DSF × Age effect, fewer areas are uniquely associated with the DSB × Age effect. These include a cluster within the bilateral lingual gyrus and cuneus (in DC) and a cluster within the right lateral PFC (in fALFF). Interestingly, although the bilateral lingual gyrus and cuneus cluster was commonly involved in both DSF × Age and DSB × Age (in DC), the effects are in opposite direction.

**Fig. 5:**
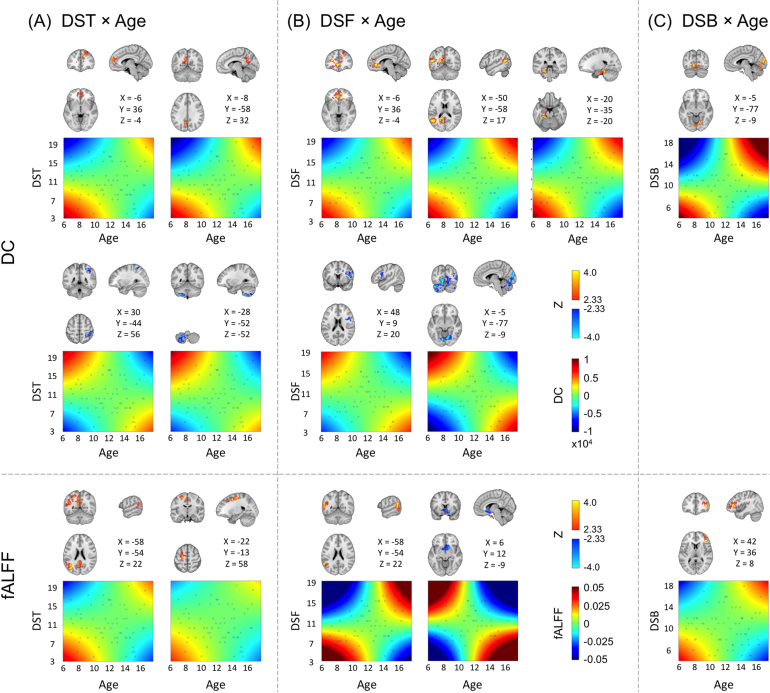
Exemplar clusters illustrating patterns of interaction with age: Digit Span total (DST) × Age (Panel A), DS forward (DSF) × Age (Panel B), and DS backward (DSB) × Age (Panel C). The whole list of clusters is reported in Table 3. Cluster locations are presented in slice view (*X*, *Y*, *Z* indicated in MNI coordinates). The magnitudes and directions of *Z*-scores are represented by either warm or cold colors. For a given cluster, the value of either Degree Centrality (DC) or fractional Amplitude of Low-Frequency Fluctuations (fALFF) is projected as a function of age and digit span score in a matrix to show how the association between intrinsic brain index and behavioral performance differed with age. The horizontal axes of the matrices represent age and the vertical axes represent DS scores. The color within each matrix represents the fitted value of the derivative score predicted by a given pair of age and DS score. The blue circles within the matrix are plotted at the intersections between observed DS scores and ages. (For interpretation of the references to color in this figure legend, the reader is referred to the web version of the article.)

To understand the nature of these interactions, the predicted derivatives were computed and plotted as a function of age and DS scores. Overall, the interaction follows one of two patterns: (1) a negative brain–behavior relationship in younger participants (e.g., a higher DST score associated with a lower DC) gradually decreases in strength and then reverses to positive at older ages (e.g., a higher DST score was associated with a higher DC); (2) a positive brain–behavior relationship followed by fading and reversal to negative at older ages. Depending on the derivative and the area, the reversal of brain–behavior relationships occurred at different ages. See Fig. 5 for exemplar clusters illustrating patterns of interaction with age.

#### 3.2.3 Secondary analyses

**Fig. 6:**
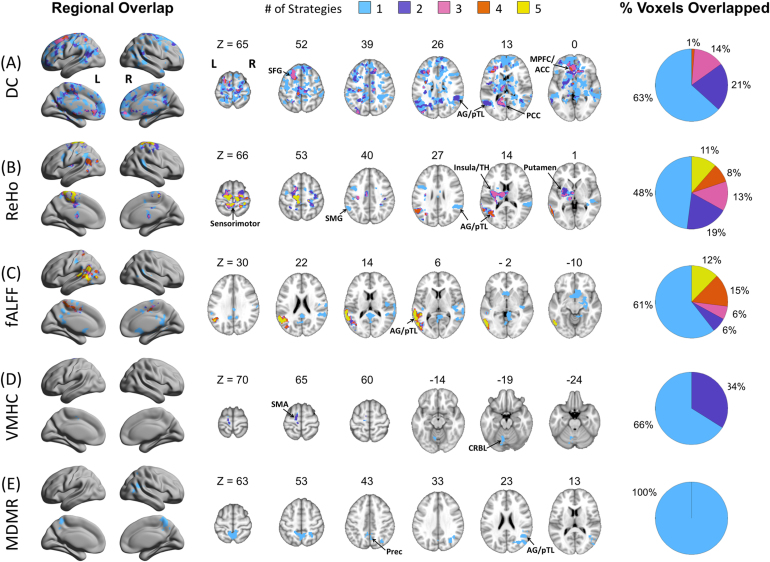
Regional overlap between nuisance correction strategies for the DSF × Age effect. Surface and slice maps are shown to depict the extent of spatial overlap between preprocessing strategies for the DSF × Age effect for each data-driven approach: Panel A–D: univariate approaches, including Degree Centrality (DC), Regional Homogeneity (ReHo), fractional Amplitude of Low-Frequency Fluctuations (fALFF), and Voxel-Mirrored Homotopic Connectivity (VMHC); Panel E: multivariate approach, Multivariate Distance Matrix Regression (MDMR). The regions overlapped by 1–5 strategies are color-coded using light blue, purple, pink, orange, and yellow, respectively. Locations of the axial (*Z*) slices are indicated in MNI coordinates. In the rightmost column (% Voxels Overlapped), the whole pie represents the total number of voxels identified by any of the 5 strategies as implicated in the DSF × Age effect. The percentage of voxels significant in 1–5 strategies is listed and shown using the same color-coding. L: left; R: right; SPG: superior frontal gyrus; AG/pTL: angular gyrus/posterior temporal lobe; PCC: posterior cingulate cortex; MPFC/ACC: medial prefrontal gyrus/anterior cingulate gyrus; SMG: supramarginal gyrus; TH: thalamus; SMA: supplementary motor area; CRBL: cerebellum; Prec: precuneus. (For interpretation of the references to color in this figure legend, the reader is referred to the web version of the article.)

To evaluate the robustness of our findings to nuisance correction strategies, we repeated our analyses employing other commonly used strategies. Because the DSF × Age effect was surprisingly more robust than the DSB × Age effect while being less explored in the literature, we used this effect to illustrate how preprocessing decisions may influence our results. The regional overlap and percent of voxels overlapping across these strategies are shown in Fig. 6. Overall, the robustness of our findings varied by processing strategies for all approaches. ReHo and fALFF were less influenced by nuisance correction strategies. In contrast, DC, VMHC, and MDMR were more influenced. The areas commonly identified by all five strategies included the bilateral sensorimotor cortex (in ReHo) and the left AG/posterior temporal lobe/lateral occipital cortex (in fALFF). For detailed description of areas detected by different number of strategies, see Supplementary Materials.

**Fig. 7:**
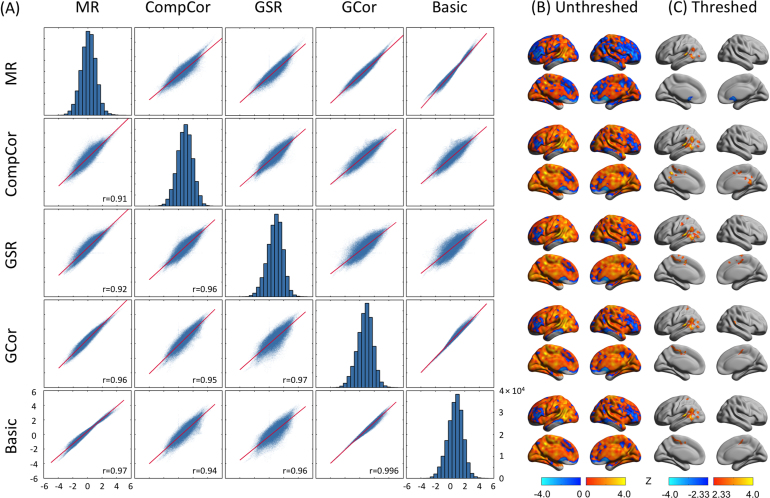
Impact of preprocessing strategies on the DSF × Age effect: fractional Amplitude of Low-Frequency Fluctuations (fALFF) approach. Panel A: Z scores of all voxels for each pair of the 5 preprocessing strategies (MR: mean regression; CompCor: component-based correction; GSR: global signal regression; GCor: global correlation correction; Basic: white matter and cerebrospinal fluid correction) scatterplots of the Z scores of all voxels were plotted to show correlations between DSF x Age effects obtained with different preprocessing strategies. The red line in the scatterplot represents the best least square fit of the *Z* scores. *r*: Pearson's correlation coefficient. The histogram of the *Z* scores of each preprocessing strategy is plotted in the diagonals. The unthresholded (unthreshed) and thresholded (threshed) *Z* statistical maps for each strategy are plotted onto an MNI space surface map in Panel B and Panel C, respectively. (For interpretation of the references to color in this figure legend, the reader is referred to the web version of the article.)

**Fig. 8:**
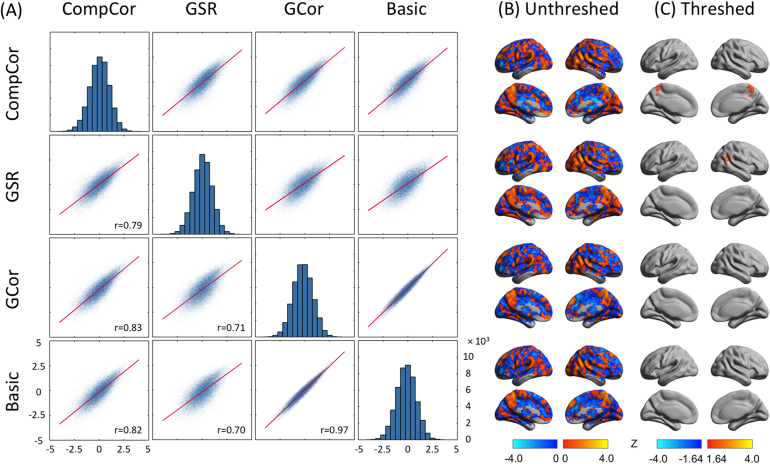
Impact of preprocessing strategies on the DSF × Age effect: Multivariate Distance Matrix Regression (MDMR) approach. The figure layout is the same as Fig. 7. The only difference is that the mean regression (MR) strategy is not applicable to this approach.

To test the relationship between results obtained using different strategies, we computed Spearman's correlation between the unthresholded *Z* statistical maps of pairs of strategies (see Fig. 7, Fig. 8 and Supplementary Figs. 2–4). Across all approaches, MR, GCor, and the basic strategy produced highly similar results indicated by high correlations among these strategies (r ranged from 0.96 to ∼1.00). The results obtained using these strategies were also highly correlated with compCor results, though at a lower magnitude (*r* ranged from 0.59 to 0.95). In contrast, the GSR results were the least consistent with those of the other strategies, especially for DC (*r* ranged from 0.24 to 0.30). This is probably because GSR biased the whole brain correlation distribution (Yan et al., 2013b), which in turn affects the total number of nodes included in the computation for a graph when correlation or probability threshold were applied.

## 4 Discussion

The present work used intrinsic brain indices derived from R-fMRI data in a pediatric sample to dissect the neural correlates of verbal WM components, as measured using the digit span task. Distinct neural mechanisms were identified for forward and backward digit span subtest performance scores, reflecting their unique demands. Importantly, we found age-dependencies in the patterns associated with each of the two subtests that involved more than just the “usual suspects” (e.g., the frontoparietal circuit) – this is particularly true for DSF scores. Before discussing neurodevelopmental insights gained from the present study, we first address the novel insights into the neural correlates of the digit span task more broadly.

### 4.1 Neural correlates of DSF and DSB: Insights from R-fMRI

Although the findings of the present work support the notion that distinct neural systems subserve the two tasks (Sun et al., 2005), a more complex picture is suggested. For the more cognitively demanding DSB subtest, we found several regions involved in attention and cognitive control associated with performance. These include the right DLPFC, the FEF, the frontal operculum cortex, anterior insular cortex, and the dACC. Additionally, the DST index suggested that connectivity of Broca's area with its right hemisphere counterpart (VMHC) relate to performance of both DSB and DSF. However, when looking at DSF specifically, we found less correspondence with the “hallmark” WM regions identified by task-based neuroimaging studies. In particular, DSF showed associations with the intrinsic properties of areas such as precuneus and lateral visual areas, which are less commonly highlighted in the task-based literature. Importantly, the lack of findings for these areas does not necessarily invalidate their relevance to task performance; instead, it may suggest that their activation levels during tasks (Paulesu et al., 1993, Salmon et al., 1996, Smith et al., 1998) are greater determinants of behavioral variability than their intrinsic properties. A prior study directly linking inter-individual differences in neural activation and behavior to intrinsic brain characteristics provides an example of a situation in which distinct intrinsic and task-activation correlates of performance have been noted (Mennes et al., 2011).

### 4.2 Dorsal ACC, a key cognitive control region, played a distinctive role in DSF and DSB

Our DC findings highlighted a dACC region showing opposite brain–behavior relationships for DSF and DSB. The dACC is implicated in a variety of cognitive functions, and its involvement in WM has been observed in both task-based (Kondo et al., 2004, Nee et al., 2013, Osaka et al., 2003) and resting-state (Li et al., 2012, Mennes et al., 2011) functional imaging studies. Consistent with previous intrinsic functional connectivity studies (Li et al., 2012), we found DC within dACC was positively related to DSB performance. In contrast, DC within this area was negatively related to DSF performance. This opposite pattern of association is consistent with task-based fMRI studies showing that dACC can contribute to different functional networks and exhibit opposite activity depending on task-load (Xu et al., 2014). These findings suggest a possible cost-benefit tradeoff associated with information flow to the dACC. Specifically, individuals with greater dACC centrality are more effective in the performance of cognitively demanding tasks, though at the cost of decreased efficiency in the performance of less demanding tasks – possibly reflecting unnecessary engagement of cognitive control systems. Additional work including task-based activation approaches can be used to further explore this possibility.

### 4.3 The use of total score as an index of WM abilities is questionable

Importantly, while the clinical literature commonly combines the performance indices of the two DS subtests to provide a more general index of verbal WM abilities (Walshaw et al., 2010), the findings question the validity of this practice. The neural correlates identified by the total score only partially reflected the unique correlates of DSF, and none of the unique correlates of DSB, suggesting the brain–behavior relationships for digit span subtests are not additive. This may explain prior observations that only DSB (not DST or DSF) was capable of distinguishing between clinical subgroups (i.e., ADHD; Rosenthal et al., 2006). Thus, our results validate concerns in the behavioral literature that using DST may obscure our understanding of the underlying neural differences (Gardner, 1981, Reynolds, 1997). At a minimum, we suggest future studies employing the DST should also examine both DSF and DSB.

### 4.4 Age-dependent neural correlates of DSF and DSB: from childhood to adolescence

Different brain areas appear to exhibit distinct age-related differences in their relationship to DSF relative to DSB, suggesting unique developmental contributions. For DSB, bilateral lingual gyrus (important for visual identification and recognition of words; Mechelli et al., 2000), occipital pole (implicated in visual imagery; Kosslyn et al., 1999), and right VLPFC (primarily responsible for retrieval of spatial information and organization of responses; Stern et al., 2000) appear to be associated with neurodevelopment of verbal WM manipulation. Compared to DSB, the age-related brain–behavior relationship differences are more robust for DSF and involved a broad array of areas spanning the ventral attention, default, somatomotor, and limbic networks. This finding is contrary to our expectation but consistent with prior structural MRI studies in children and adolescents (Ostby et al., 2011, Rossi et al., 2013). These studies found that age-related changes in associations between cortical thickness and WM were only for the storage but not the executive component of WM. The greater spatial extent of maturational differences for DSF suggests a larger change for brain areas implicated in DSF from childhood to adolescence. The ability of R-fMRI derivatives to index inter-individual differences in digit span performance and capture age-related differences in these indices emphasizes the potential utility of these tools for investigating developmental problems.

### 4.5 New foci contributing to the development of WM maintenance: Angular gyrus and subcallosum

Three approaches (i.e., DC, ReHo, fALFF) converge on a cluster composed of left angular gyrus extending into posterior middle temporal gyrus, as a locus potentially contributing to the neurodevelopment of WM maintenance. Moreover, this cluster appeared to be robust to preprocessing decisions (e.g., significant in all five strategies for fALFF and in four strategies for ReHo). These convergences suggest a pervasive link between the intrinsic features of this area and WM development. Specifically, a negative relationship between DSF and intrinsic functional properties was observed in younger children, which gradually faded and then reversed at older ages to positive. This suggests that the functional relevance of AG to verbal WM storage is developmentally sensitive, which is consistent with task-based fMRI studies showing age-related differences in its involvement in verbal WM for words (Church et al., 2008). As the left AG has been implicated in digit perception, semantic processing, verbal coding of numbers, and storage of verbal materials (Seghier, 2013), changes in these processes may contribute to the development of verbal WM maintenance. Our results linking AG to verbal WM development may facilitate our understanding of learning impairments, given that verbal WM is core to children's academic abilities, and that the involvement of AG in learning abilities/disability has been well documented (Butterworth, 2010, Shaywitz et al., 2002).

Other areas highlighted by at least two approaches included sensorimotor cortex, the medial temporal lobe (MTL), subcallosum extending into accumbens, and cerebellum. Except for subcallosum, the other areas have been previously implicated in the age-related differences in WM. For example, van den Bosch et al. (2014) reported greater connectivity in adolescents relative to children within a network involving left motor area and right cerebellum during the encoding phase of WM maintenance. The MTL is implicated in memory encoding and retrieval (Eichenbaum et al., 2007), with its putative contributions to WM maintenance decreasing from early to late adolescence (Finn et al., 2010).

More importantly, the current study identified the involvement of subcallosal cortex/accumbens, an area largely overlooked in verbal WM development. This deep limbic region is implicated in reward, motivational, and emotional processing (Hamani et al., 2011). In NeuroSynth (Yarkoni et al., 2011), a ‘reverse inference’ at coordinates [*x* = 4; *y* = 8; *z* = −10] revealed the following top associated features for this area: ventral striatum, reward, nucleus accumbens, accumbens, striatum, motivation, subgenual, reward anticipation, and dopamine. One prior study has suggested involvement of subcallosal gyrus in spatial WM development using task-based fMRI (Nagel et al., 2005). The current results extend its role to verbal WM maintenance. As subcallosum/nucleus accumbens/striatum is commonly involved in a broad range of developmental psychiatric disorders, including autism (Di Martino et al., 2011) and depression (Hamani et al., 2011), future work would benefit from fine-grained mapping of its function in brain development.

### 4.6 Validation of the DSF × Age effect using different preprocessing strategies

A key challenge of R-fMRI, and arguably fMRI more broadly, is the need to make preprocessing decisions that can impact our ability to detect findings. While we used what we believed to be an optimal set of decisions, we examined the dependences of our findings on preprocessing strategy decisions, using the DSF × Age interaction. First we compared three processing strategies only differing with respect to group-level correction for nuisance signals: MR (global mean + mean FD, our primary analytic approach), GCor (global correlation + mean FD), and basic model (mean FD alone). We found that the results obtained using MR were highly correlated with the other two strategies. Next, we compared MR to two strategies that attempt to correct for nuisance signals differently at the individual subject level: CompCor and GSR. Results obtained with these two strategies were impressively similar to those obtained with MR, though the extent of similarity is less compared to GCor and the basic model. Among all approaches, GSR was the least consistent with other strategies. One noteworthy caveat is that while the overall pattern of findings obtained across the brain using different strategies was consistent (except for DC), applying thresholds and stringent multiple comparison corrections to the maps can yield notable differences for certain derivatives. Such differences may reflect variations that can push near-threshold findings slightly above or below criteria for significance.

### 4.7 Limitations

We note a number of limitations. First, cross-sectional findings cannot be treated as definitive because of potential differences among age-cohorts. Our cross-sectional age-related findings should motivate definitive examinations using longitudinal study designs, ideally starting from an earlier age (Di Martino et al., 2014). Second, although digit span is a commonly used and standardized task, it is warranted to replicate this work using other clinically validated and standardized tasks that differentially vary maintenance and manipulation components of WM (e.g., Automated Working Memory Assessment Battery; Alloway, 2011); Third, across participants, the specific eyes open/closed status varied; while several studies have shown that intrinsic brain activity differs between eyes open and closed states (Liu et al., 2013, Yan et al., 2009), this factor is unlikely to confound our age-related findings, as it did not differ between our children and adolescent groups. Nonetheless, we repeated our univariate group analyses with this factor included as a nuisance variable, finding highly similar results (see Supplementary Fig. 6).

Fourth, a challenge inherent to any neurodevelopmental study is head motion. To avoid potentially artifactual findings in the present work, we corrected for motion at both the individual and group levels; in addition we validated our findings using different nuisance correction strategies. Fifth, we used multiple static approaches to measure relative stable properties of spontaneous brain activity at a single timepoint. With development of dynamic intrinsic functional connectivity methodologies, recent studies have begun to link dynamic changes in macroscopic neural activity patterns to cognition and behavior (e.g., Hutchison et al., 2013, Yang et al., 2014). Future work with longer recordings, including multiple sessions, will allow us to evaluate the reproducibility and reliability of the static brain–behavioral relationship and perform dynamic analyses to delineate the association between dynamic features and working memory development.

Sixth, the functional significance of having an association detectable with multiple indices as opposed to one is unclear, given the speculative nature of their underlying physiology. Each R-fMRI derivative is believed to assess a distinctive aspect of intrinsic brain function, although they are not entirely independent of one another. For a given region, when a brain–behavioral relationship emerges for more than one R-fMRI measure, it may suggest a more profound involvement of this region. Future work clarifying the underlying neural basis of the various measures remains essential. Finally, it is important to keep in mind that associations between R-fMRI measures and digit span task performance do not support inferring mechanisms of verbal working memory; arguably, a number of cautions arise regarding potential limitations in the establishment of mechanism from task fMRI as well. Future work using manipulation approaches such as transcranial magnetic stimulation may help push closer to the understanding of mechanisms.

### 4.8 Conclusion

We systematically explored intrinsic functional brain indices of verbal WM performance and identified the brain–behavior associations that are modulated by age in a cross-sectional pediatric sample. Regardless of age, DSB performance was uniquely related to intrinsic features of regions belonging to commonly reported WM circuits, while the unique neural correlates of DSF performance include areas less commonly implicated in the storage component of verbal WM (e.g., precuneus and lateral visual areas). Compared to DSB, the age-related brain–behavior relationship changes are more robust for DSF and involve a broader range of networks (ventral attention, default, somatomotor, limbic networks). These include a number of areas not commonly associated with verbal WM (e.g., angular gyrus and subcallosum). Taken together, these results underscore the importance of examining the neural correlates of verbal WM from a developmental perspective along with the need for greater consideration of regions beyond the “known” correlates of verbal WM.

## Conflict of interest

All authors declared no conflict of interest.
