# Supplementary material for: Intrinsic brain indices of verbal working memory capacity in children and adolescents
Source: Dev Cogn Neurosci. 2015 Aug 4;15:67–82. doi: 10.1016/j.dcn.2015.07.007 (PMC4696540; doi:10.1016/j.dcn.2015.07.007)

**Supplementary Results**

*Secondary analysis: areas exhibiting a significant Digit Span Forward (DSF)×Age interaction identified by different strategies*

For Degree Centrality (DC: **Fig. 6A**), no regions were commonly identified by all five preprocessing strategies as exhibiting a significant DSF×Age interaction. Only 1% of the voxels out of all voxels significant in any of the five preprocessing strategies were commonly identified by four strategies and these voxels were scattered across the brain. Three strategies commonly identified 14% of the voxels and these voxels located in the left superior frontal gyrus (SFG), bilateral medial prefrontal cortex/anterior cingulate cortex (MPFC/ACC), left middle temporal gyrus, posterior cingulate gyrus (PCC), bilateral lingual gyrus, bilateral cerebellum, and left subcortical regions. Two strategies overlap in 21% of voxels located within the prefrontal cortex, bilateral angular gyrus/posterior temporal lobe (AG/pTL), and bilateral PCC/precuneus. Most of the findings (63%) are identified by a single strategy and involve large-scale brain regions scattered over the whole-brain including the four main cortical lobes, cerebellum, and subcortical regions.

For Regional Homogeneity (ReHo: **Fig. 6B**), 11% of the voxels are robust to all five preprocessing strategies and mainly located in the bilateral sensorimotor cortex. 8% are significant in four strategies and mainly located in the AG/pTL. 13% of the results are robust to three strategies; these included the left insula and thalamus. 19% are significant in two strategies and located within the bilateral sensorimotor cortex and left putamen. 48% are detected by a single strategy and within the left precentral gyrus, left lateral and medial temporal lobe, bilateral temporoparietal junction (TPJ), subcortical regions, and left cerebellum.

For fractional Amplitude of Low-Frequency Fluctuations (fALFF: **Fig. 6C**), 12% of results are detected by all five strategies and are located within the left AG/pTL extending into the lateral occipital cortex (LOC). 15% of regions are significant in four strategies; they comprise the AG/pTL/LOC and the bilateral sensorimotor cortex. The areas identified by two or three strategies are also around these two regions and included 12% of the significant results. 61% of the results are identified by a single strategy and include the bilateral PCC/precuneous, right TPJ, and bilateral subcallosal cortex/nucleus accumbens.

For Voxel-Mirrored Homotopic Connectivity (VMHC: **Fig. 6D**), the DSF×Age interaction was only observed within a small portion of the supplementary-motor area and 34% of this region was robust to 2 strategies (i.e., GSR and GCor). For the Multivariate Distance Matrix Regression (MDMR) – based approach (**Fig. 6E**), no regions showing significant DSF×Age interaction are detected by more than 2 strategies. The regions detected by a single strategy include bilateral precuneus (CompCor) and the right AG/pTL/LOC (GSR).

**Supplementary Table 1.** MDMR-guided intrinsic function connectivity analyses

| **Effect** | **Seed ROI** | **Target ROI** | **BA** | **Network (Yeo et al., 2011)** | **Center of Mass (MNI)** | | | **Volume (# Voxels)** |
| --- | --- | --- | --- | --- | --- | --- | --- | --- |
|  |  |  |  |  | **X** | **Y** | **Z** |  |
| **DST** | B Precuneus extending into R LOC | L Frontal pole/SFG/  Paracingulate gyrus | 10/32 | Default | -5 | 57 | 17 | 182 |
| **DSF** | B Precuneus extending into L LOC | R SPL/Precuneus/  LOC | 5/7 | DorsAttn | 20 | -57 | 60 | 225 |
| **DSB** | L SFG/MFG | L STG/MTG | 20/21 | Default | -54 | -21 | -10 | 239 |
|  |  | B PCC/Precuneus | 23 | Default | -1 | -52 | 31 | 631 |
|  |  | B Frontal pole | 9/10/11 | Default | 0 | 50 | 17 | 1001 |
|  |  | B ACC/Para-cingulate gyrus | 24/32 | Control/  VentAttn | 3 | 20 | 36 | 441 |
|  |  | L Frontal pole/MFG/Insula | 45/46/48 | Control/  VentAttn | -38 | 25 | 13 | 496 |
|  |  | R Frontal pole/  Frontal operculum cortex/Insula | 45/46/47/48 | Control/  VentAttn | 42 | 27 | 9 | 836 |
| **DST X Age** | B SPL/  Postcentral gyrus and Precuneus | L pre- and post-central gyrus, L SPL | 2/3/4/40 | SomMot/  DorsAttn | -45 | -26 | 48 | 264 |
|  |  | R pre- and post-central gyrus, R LOC | 2/3/4/7 | SomMot/DorsAttn | 42 | -30 | 47 | 753 |

Note: DST = digit span total score; DSF = digit span forward; DSB = digit span backward; ROI = region of interest; B = bilateral; R = right; LOC = lateral occipital cortex; L = left; SFG = superior frontal gyrus; MFG = middle frontal gyrus; SPL = superior parietal lobule; STG = superior temporal gyrus; MTG = middle temporal gyrus; PCC = posterior cingulate cortex; ACC = Anterior cingulate cortex; MFG = middle frontal gyrus; BA = Brodmann Area; DorsAttn = dorsal attention network; Control = frontoparietal control network; VentAttn = ventral attention network; SomMot = somatomotor network; voxel size = 3 x 3 x 3 mm.

**Supplementary Figure 1:** Age distribution (N=68, mean = 12.13 ± 2.74 years)


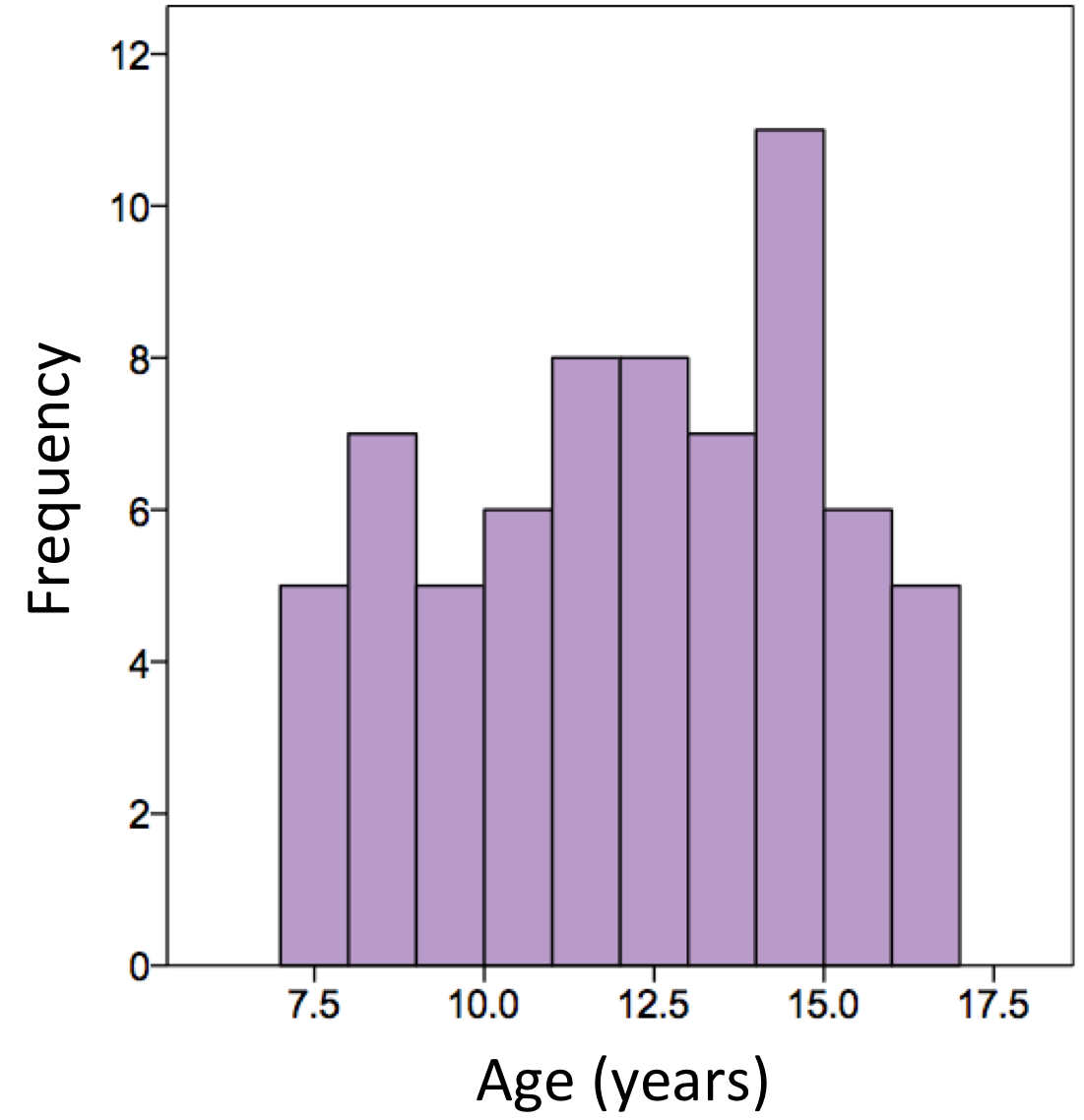


**Supplementary Figure 2:** *Impact of preprocessing strategies on the DSF × Age effect: Degree Centrality (DC).* The figure layout is the same as Figure 7. MR: mean regression; CompCor: component-based correction; GSR: global signal regression; GCor: global correlation correction; Basic: white matter and cerebrospinal fluid correction.


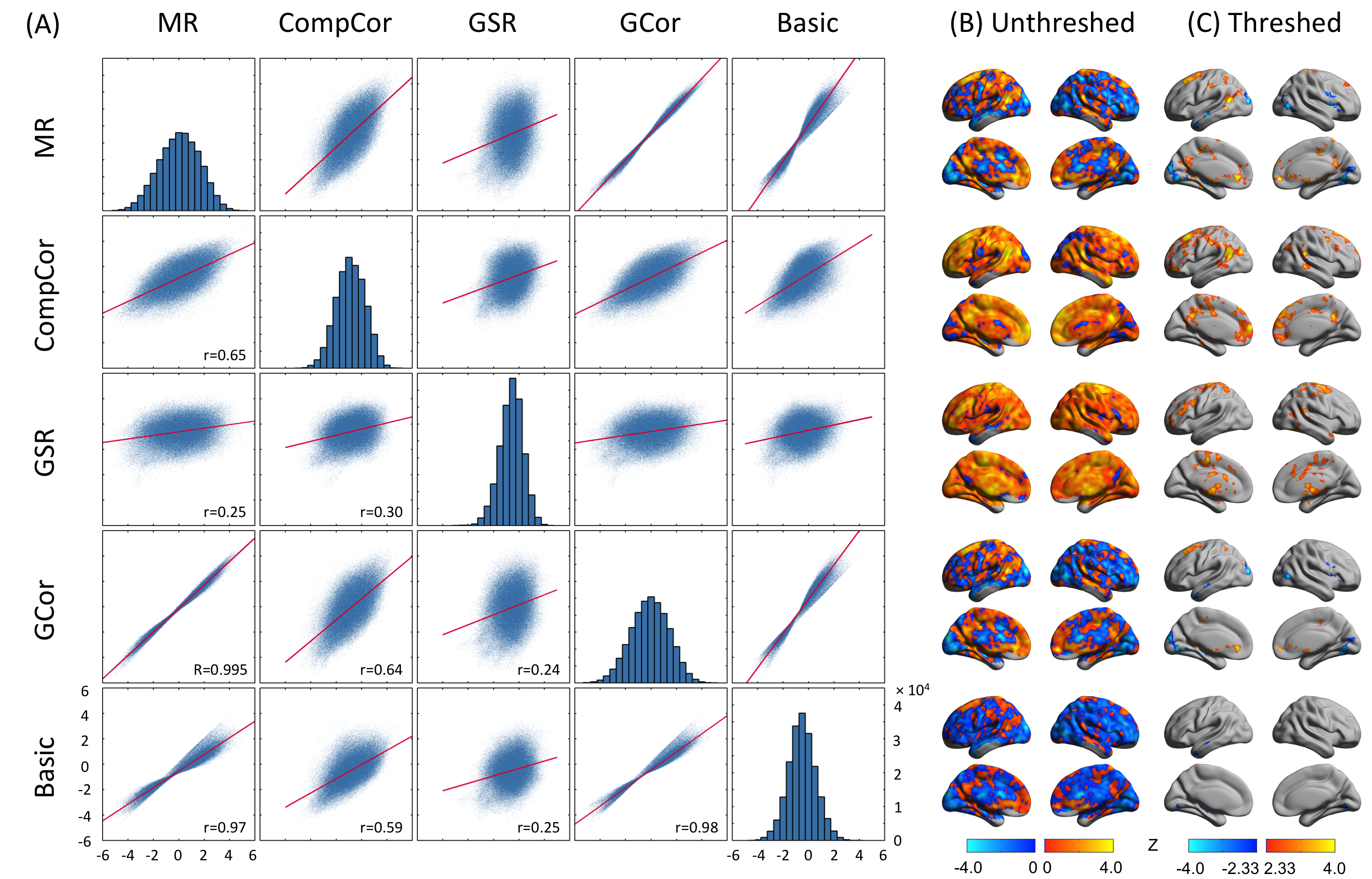


**Supplementary Figure 3:** *Impact of preprocessing strategies on the DSF×Age effect: Regional Homogeneity (ReHo).* The figure layout is the same as Figure 7. MR: mean regression; CompCor: component-based correction; GSR: global signal regression; GCor: global correlation correction; Basic: white matter and cerebrospinal fluid correction.


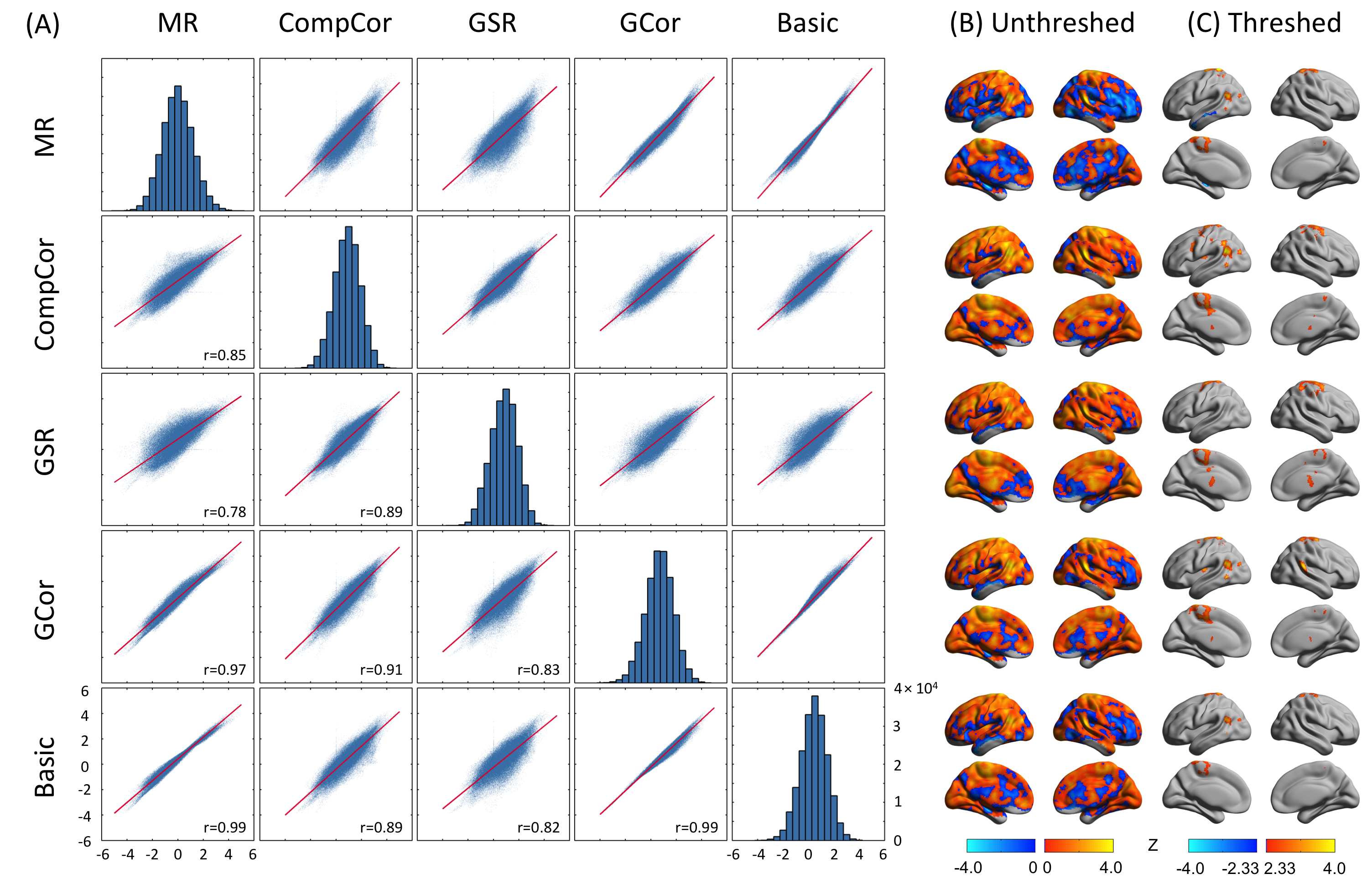


**Supplementary Figure 4:** *Impact of preprocessing strategies on the DSF×Age effect: Voxel-Mirrored Homotopic Connectivity (VMHC).* The figure layout is the same as Figure 7. MR: mean regression; CompCor: component-based correction; GSR: global signal regression; GCor: global correlation correction; Basic: white matter and cerebrospinal fluid correction.


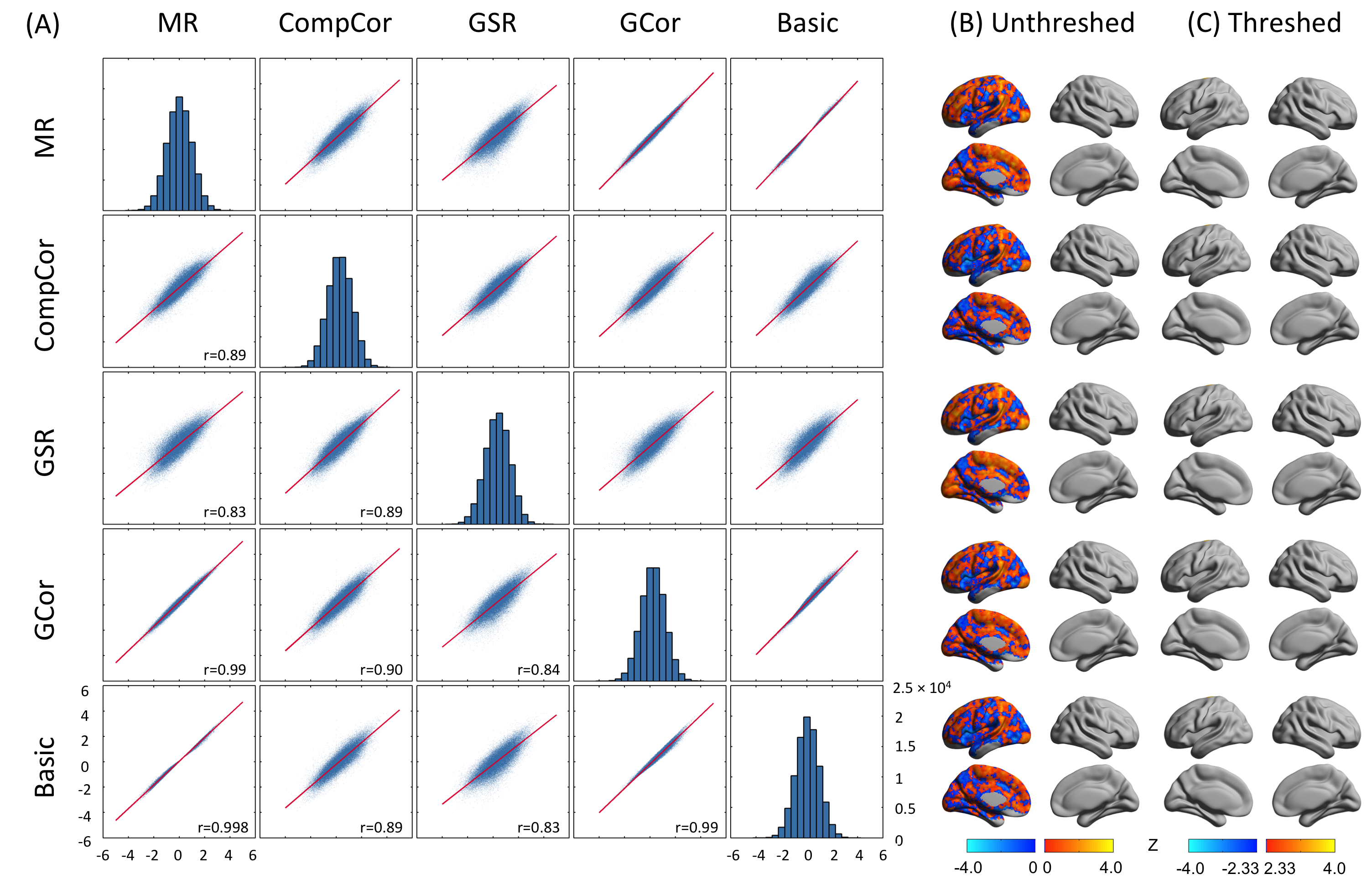


**Supplementary Figure 5:** Univariate results corrected (Z>2.33, p<0.0125; left Panel) and uncorrected (Z>2.33, p<0.05; right Panel) for four approaches.


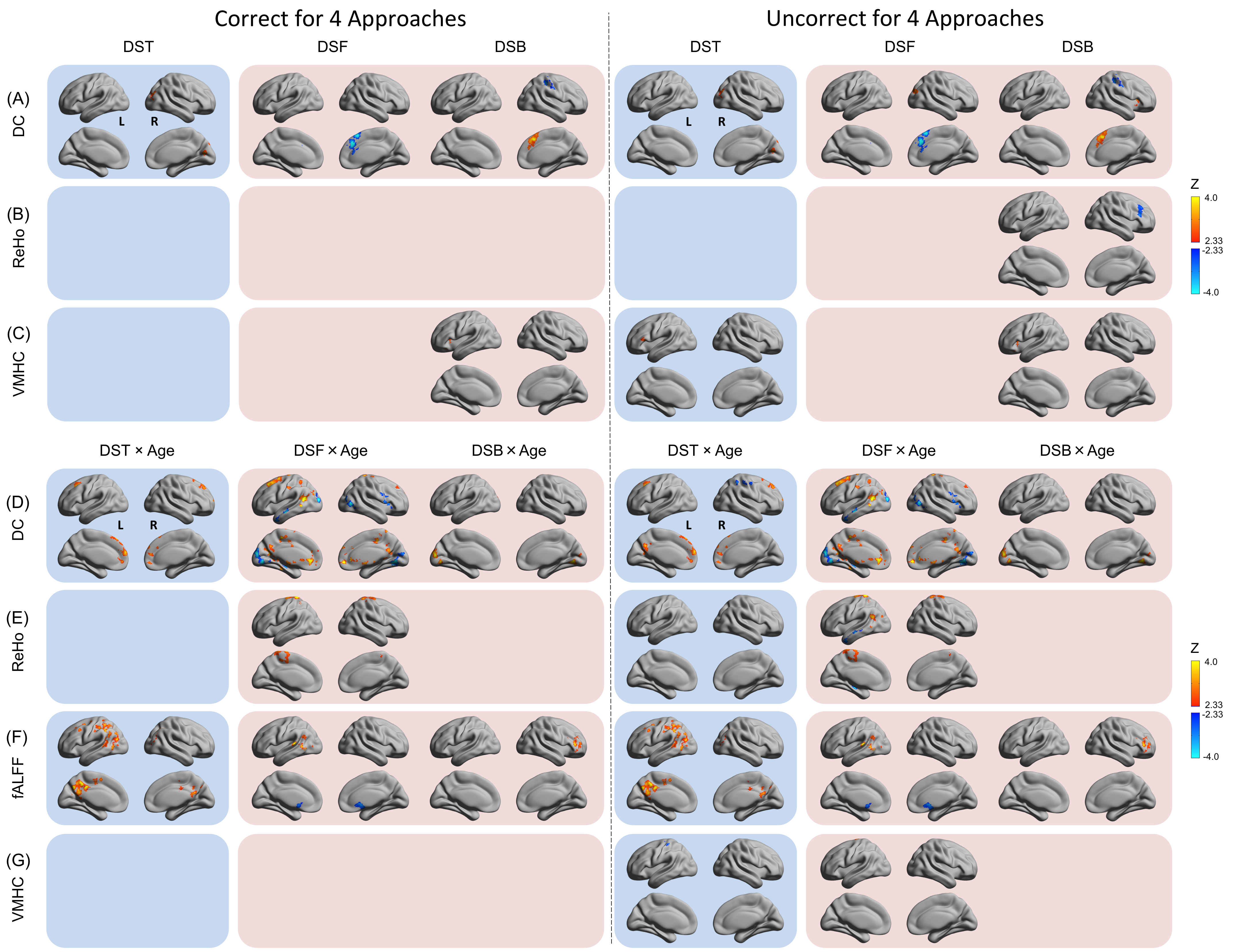


**Supplementary Figure 6:** Univariate results obtained with eye condition (eyes open vs. eyes closed) included as a nuisance covariate.


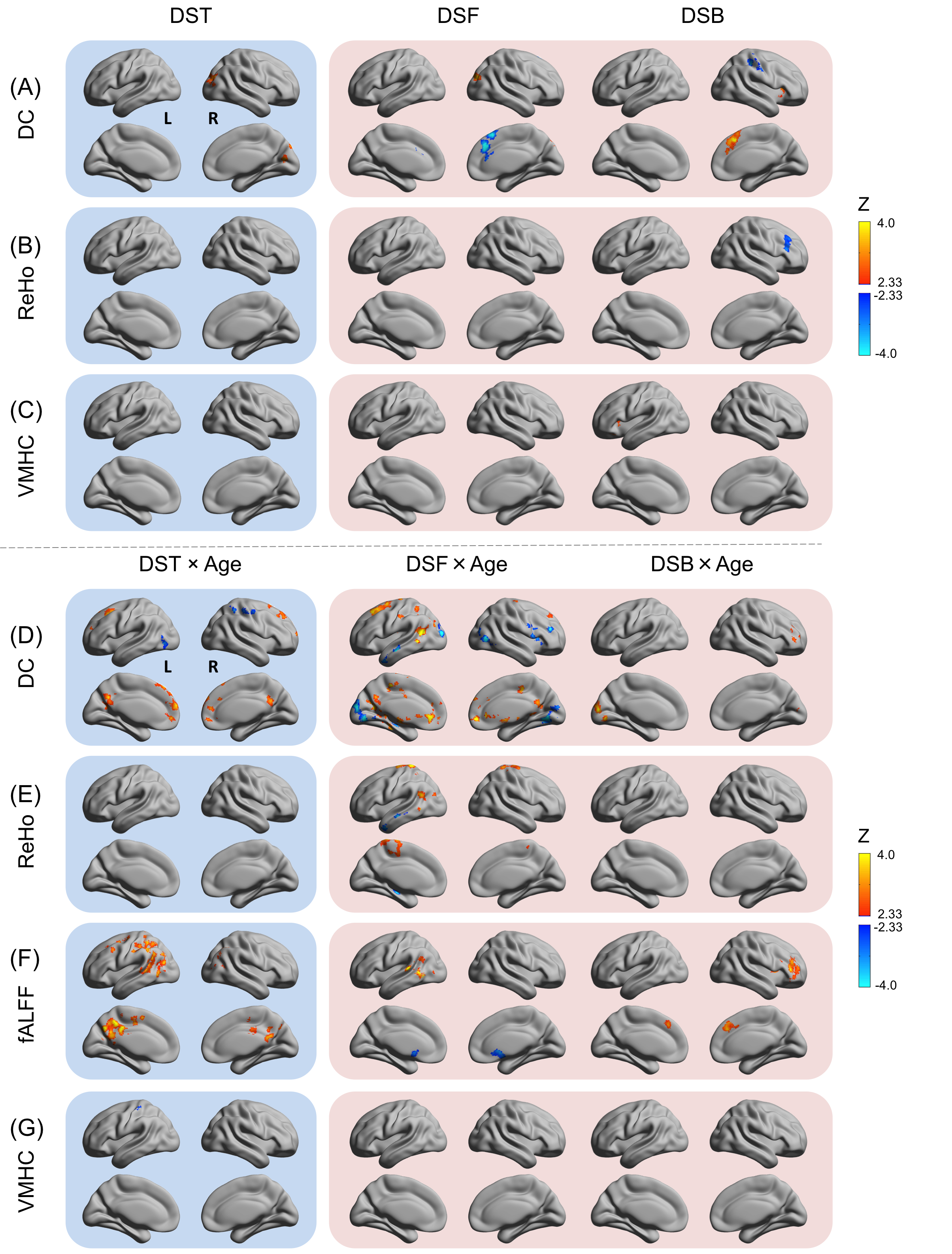

Supplement: Supplementary file 1 [file mmc1.docx]
